# Ferric Carboxymaltose and Erythropoiesis-Stimulating Agent Treatment Reduces the Rate of Blood Transfusion in Refractory Anemia

**DOI:** 10.3390/jcm11164744

**Published:** 2022-08-14

**Authors:** Antonio Gidaro, Alessandro Palmerio Delitala, Alessandra Berzuini, Mark J. Soloski, Pietro Manca, Dante Castro, Emanuele Salvi, Roberto Manetti, Giorgio Lambertenghi Deliliers, Roberto Castelli

**Affiliations:** 1 Department of Biomedical and Clinical Sciences Luigi Sacco, Luigi Sacco Hospital, University of Milan, Via G.B. Grassi N° 74, 20157 Milan, Italy; 2 Department of Medicine, Surgery and Pharmacy, University of Sassari, Viale San Pietro N° 8, 07100 Sassari, Italy; 3 Fondazione IRCCS Ca’ Granda Ospedale Maggiore Policlinico, Via Francesco Sforza, 28, 20122 Milan, Italy; 4 Division of Rheumatology, Johns Hopkins University School of Medicine, Baltimore, MD 21224, USA; 5 Transfusion Center, Azienda Ospedaliero Universitaria, Piazza Università, N° 21, 07100 Sassari, Italy; 6 Fondazione Mattarelli Largo della Crocetta, 2, 20122 Milan, Italy

**Keywords:** low-risk myelodysplastic syndromes, refractory anemia, erythropoiesis-stimulating agent, erythropoietin, ferric carboxymaltose, ferric gluconate, iron supplementation

## Abstract

**Background**: Erythropoiesis-stimulating agents (ESAs) are used to treat refractory anemia (RA). Guidelines suggest iron supplementation for unresponsive patients, regardless of iron deficiency. The primary aim of this study was to evaluate the effect of iron supplementation with ferric carboxymaltose (FCM) on the reduction of red blood cell transfusion (RBCT) rate in transfusion-dependent RA patients. **Methods**: This was a prospective quasi-randomized study, wherein patients were randomly assigned into three groups: (A) ESAs alone, (B) ferric gluconate (FG) and ESAs, and (C) FCM and ESAs. Hemoglobin and ferritin levels, as well as the number of RBCTs at 4 and 28 weeks were compared. Economic evaluation was also performed. **Results**: A total of 113 RA patients were enrolled. In total, 43 were treated with intravenous FG and ESAs, 38 with FCM and ESAs, and 32 with ESAs alone. At both follow-ups, erythropoietic response was increased in those receiving iron as compared with those with ESAs alone (*p* = 0.001), regardless of the type of iron. At one month, ferritin levels were higher in the FCM and ESA groups (*p* = 0.001). RBCTs were lower in both iron groups. The less costly treatment strategy was FCM, followed by FG, and lastly ESAs. **Conclusions**: Addition of iron to ESAs in RA reduced RBCT requirement and improved hemoglobin values.

## 1. Introduction

Myelodysplastic syndromes (MDS) are a heterogeneous group of clonal stem cell disorders characterized by various grades of peripheral cytopenia and bone marrow hyper-cellularity [1]. Incidence of MDS increases with age, with clonal hematopoiesis being strictly correlated with age, especially in those older than 65 years. Risk stratification, which impacts on prognosis, survival, and progression to acute leukemia, is largely linked with the International Prognostic Scoring System (IPSS) along with molecular testing [2]. High-risk patients have poor cytogenetic testing, with aberrant or complex karyotypes and higher levels of blasts cells. Clinically, this is an aggressive disease, behaving similarly to acute myeloid leukemia (AML). Treatment with hypomethylating agents is aimed towards slow progression to AML and should be started as soon as possible [3]. On the other hand, 90% of low-risk MDS experience anemia, which can lead to symptoms of fatigue, cardiac morbidity, and cognitive impairment [3]. Treatments of low-risk MDS are largely aimed to mitigate symptoms of anemia, reduce cardiovascular events, and improve quality of life [4]. Specifically, 40% of patients are transfusion dependent, needing regular red blood cell (RBC) transfusions as part of their supportive care therapy.

It is reasonable to suspect that transfusion-dependent MDS patients identify a subset of patients in which clonal hematopoiesis is largely linked with progression to AML. Nevertheless, long-term dependence on transfusion treatment is linked with poor outcome due to cardiac events, including hepatic and cardiac iron overload [5,6] and non-leukemic death. Treatment with transfusion provides transient relief in anemia-related symptoms, improving the quality of life [7]. Erythropoiesis-stimulating agents (ESA) should be avoided in patients with MDS and elevated baseline erythropoietin (EPO) levels (>500 IU/L). Indeed, some studies suggest that those patients are unlikely to respond to ESA therapy [8,9]. Furthermore, a recent study has suggested that even lower baseline EPO levels (<200 IU/L) are associated with a better hemoglobin (Hb) response to ESA supplementation [10]. Lower pretreatment red blood cell transfusion (RBCT) dependence (<2 units per month) has also been associated with a higher likelihood of ESA response in patients with MDS [10]. About 40–50% of patients treated with ESAs have an erythroid response lasting about 24 months [11]. The use of ESAs to manage anemia raises Hb levels and reduces the need for RBCT but increases the risk of thromboembolic events [12]. For these reasons, clinicians should carefully evaluate the thrombotic risk for each patient before prescribing ESAs. Iron replacement may be used to improve Hb response and reduce RBCT for patients receiving ESAs with or without iron deficiency [6]. Ferric carboxymaltose (FCM), a novel iron complex that consists of a ferric hydroxide core stabilized by a carbohydrate shell, allows for controlled delivery of iron to target tissues. Administered intravenously, it is effective in the treatment of iron deficiency anemia, delivering a replenishment dose of up to 1000 mg of iron during a minimum administration time of </= 15 min [13,14,15,16,17]. Results of several randomized trials have shown that intravenously administered FCM rapidly improves hemoglobin levels and replenishes depleted iron stores in various populations of patients with iron deficiency anemia, including those with inflammatory bowel disease [13], heavy uterine bleeding [14], postpartum iron deficiency anemia [15], heart failure [16], and chronic kidney disease [17]. It is also well tolerated in clinical trials [13,14,15,16,17]. FCM is, therefore, an effective option in the treatment of iron deficiency anemia in patients for whom oral iron preparations are ineffective or cannot be administered.

The aim of this study was to assess whether the use of intravenous FCM can be of benefit to improve hemoglobin levels and reduce the need of blood transfusion in MDS anemic patients treated with ESAs. Secondary aims were assessing the ferritin level of patients and performing an economic evaluation.

## 2. Materials and Methods

This was a prospective quasi-randomized monocentric study performed in patients with low-risk MDS refractory anemia (RA). Alongside this was a cost-effectiveness assessment within a process of quality improvement and resource maximization.

MDS patients were enrolled from 30 March 2013 to 30 March 2021. The study was approved by the Ethical Committee of Human Experimentation (no. 206, date 29 January 2013) and was conducted in accordance with the Declaration of Helsinki. All patients provided informed consent, and a local ethical committee approved the study.

The inclusion criteria were (1) low-risk MDS (IPSS scale score = 0); (2) Hb levels under 9.5 mg/dL; (3) at least one RBC transfusion in the last three months of pretreatment; and (4) age over 65 years.

The exclusion criteria were (1) baseline erythropoietin levels > 500 IU/L; (2) pretreatment RBC transfusion dependence (>2 units per month); and (3) hemosiderosis with ferritin > 1000 ng/mL.

Hospital therapy’s guideline of low-risk MDS has changed over time. For these reasons, patients were quasi-randomized into three groups according to time of enrollment and in-use treatment. Group (A) involved ESAs alone and were enrolled during three years from 2013 to 2015; group (B) involved intravenous iron supplementation with ferric gluconate (FG) (125 mg every week) and ESAs and were enrolled during three years from 2016 to 2018; group (C) involved intravenous FCM iron (1000 mg every four weeks) supplementation and ESAs and were enrolled for three years from 2019 to 2021. At baseline, homogeneity of the three groups was assessed.

Usually, a standard dose of two vials of FG (125 mg) was diluted in 250 mL of saline and administered in 45−60 min every week. A standard FCM dose of 1000 mg was diluted in 250 mL of saline and administered in 15–20 min in one presentation, usually every four weeks.

ESA treatment was performed according to ASH and ASCO guidelines for the treatment of anemia in MDS syndromes [8,9]. A subcutaneous injection of 40,000 UI/week was performed on patients. ESAs reduced by 25% when Hb reached a level needed to avoid transfusion or Hb increased 1 g/dL in 2 weeks. ESA dose increased to 80,000 UI/week if Hb increased by less than 1 g/dL and remained below 9 g/dL after 1 month of therapy.

Moreover, for all the patients enrolled, 7.5 mg of oral calcium levofolinate/day and 400 mg of vitamin B12 orally/day were administered.

The transfusion of one unit of red packed cells was allowed if the Hb level was below 8 g/dL or if Hb level was below 9 g/dL with a grade 3–4 organ failure or if anemia-related symptoms were considered unacceptable or dangerous for patients.

### 2.1. Clinical Parameters and Measurements

Clinical parameters considered in the evaluation included baseline general patient demographics, biochemical characteristics (Hb, ferritin), and prognostic disease class (IPSS).

The first set of aims was assessing disease progression and treatment response measuring (1) number of patients with hemoglobin > 9 (g/dL) after 4 weeks (1 month); (2) number of patients with hemoglobin > 10 (g/dL) after 28 weeks (6 months); and (3) number of transfusions during follow-up time.

Secondary aims were (1) number of patients with ferritin > 40 (ng/mL) after 4 weeks (1 month); (2) number of patients with ferritin > 40 (ng/mL) after 28 weeks (6 months); (3) iron supplementation’s side effect, intolerance, or development of ferritin > 1000 ng/mL; and (4) an estimate of the costs independent of the drug’s cost, but rather linked to the final outcome (efficacy) of the therapeutic strategy used during the observation period.

### 2.2. Economic Evaluation

The economic assessment was based on the calculation of expenditures of direct costs for EPO doses, RBC transfusions, blood tests, out-patient visit, and iron supplementation.

Unit costs per each item used were:

- 1 day of admission to day hospital (including nursing support and board): EUR 200;
- Cost of 1 unit of concentrated red blood cells or platelets: approximately EUR 180;
- Erythropoiesis-stimulating agent 80,000 U/I: approximately EUR 776/ampoule;
- Erythropoiesis-stimulating agent 40,000 U/I: approximately EUR 388/ampoule;
- Erythropoiesis-stimulating agent 30,000 U/I: approximately EUR 290/ampoule;
- Intravenous ferric gluconate: approximately EUR 15/dose;
- Intravenous ferric carboxymaltose: approximately EUR 272/dose.

Such costs represent a national average for Italy, based on the expenditures/reference acquisition prices provided by regional health services (for treatments, hospitalization, etc.). For each patient, we calculated an overall cost of treatment for 28 weeks (6 months) observation period. The total cost per individual patient was then divided into 4 weeks cost and one-week cost. Then, in each treatment group, the median value of treatment was calculated. This provided an estimate of the costs independent of the drug’s cost, but rather linked to the final outcome (efficacy) of the therapeutic strategy used during the observation period.

### 2.3. Statistical Analysis

The Kolmogorov–Smirnov test was used to evaluate the normality of the distribution of data. Qualitative data were expressed as numbers and percentages. Fisher’s exact tests were used for groups comparisons. Quantitative data were expressed as mean, standard deviation, or median and interquartile range. Statistical differences were investigated with repeated measures analysis of variance (rANOVA or mixed-effect analysis, as indicated), while specific differences were assessed with Holm–Bonferroni correction for multiple comparisons. A *p*-value < 0.05 was considered statistically significant.

The statistical analysis of data was performed by using SPSS (statistical package for social science-SPSS, Inc., Chicago, IL, USA, version 20).

## 3. Results

A total of 113 MDS transfusion-dependent patients affected by refractory anemia were enrolled. The median age was 70 years (IQR 66–74); 71 were men (69.5%) and 52 were women (30.5%) (Table 1). According to iron supplementation, patients were divided into three groups: 43 patients were treated with intravenous FG and ESAs (group A); 38 patients were treated with intravenous FCM supplementation and ESAs (group B); 32 patients were treated with ESAs alone (group C). The patients presented similar clinical and hematological characteristics, as shown in Table 1.

All enrolled patients reached 28 weeks (six months) of follow-up without therapy’s modification or early drop out.

**Figure 1:**
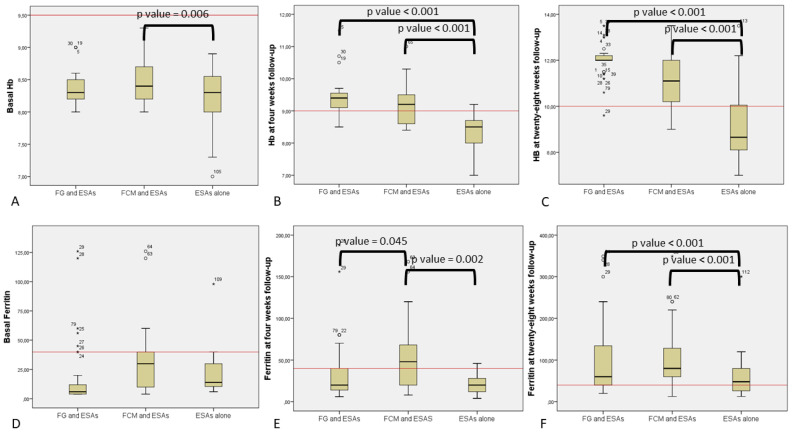
Comparison of patient’s hemoglobin and ferritin based on different treatments: (**A**) Basal hemoglobin (Hb) in ferric gluconate (FG) and erythropoiesis-stimulating agents (ESAs); ferric carboxymaltose (FCM) and ESAs; and ESAs alone. Continuous red line represents the clinical cut-off for enrollment (9.5 g/dL). (**B**) Hb at four weeks follow-up in FG and ESAs; FCM and ESAs; and ESAs alone. Continuous red line is the clinical cut-off for group comparison (9.5 g/dL). (**C**) Hb at 28 weeks of follow-up in FG and ESAs; FCM and ESAs; and ESAs alone. Continuous red line represents the clinical cut-off for group comparison (10 g/dL). (**D**) Basal ferritin in FG and ESAs; FCM and ESAs; and ESAs alone. Continuous red line represents the clinical cut-off for group comparison (40 ng/mL). (**E**) Ferritin at four weeks of follow-up in FG and ESAs; FCM and ESAs; and ESAs alone. Continuous red line represents the clinical cut-off for group comparison (40 ng/mL). (**F**) Ferritin at 28 weeks follow-up in FG and ESAs; FCM and ESAs; and ESAs alone. Continuous red line represents the clinical cut-off for group comparison (40 ng/mL).

Basal Hb was statistical higher in FCM than the ESAs group, but when using the clinical cut off of 9 g/dL to match patients, this difference disappeared (Figure 1A).

At the first follow-up at four weeks, statistical analysis showed that erythropoietic response was significantly increased in the groups of patients receiving iron supplementation as compared with ESAs alone (*p* < 0001), regardless of the type of iron supplementation (Figure 1B). This increase was confirmed even using the clinical cut off value and indicates a higher number of patients with more than 9 g/dL Hb in groups A and B.

The more effective recovery for both iron supplementation groups (B and C) was also apparent at the 28 weeks follow-up (Figure 1C). At this time point, FG had higher absolute Hb levels when compared to FCM, but no difference was found using the clinical cut-off (10 g/dL).

The number of transfusions were lower in both iron supplemented groups compared to those supplemented with ESAs alone, and this was independent of the follow-up time (4 vs. 28 weeks). Patients were transfused only in the four weeks of follow-up in both iron supplementation groups. The FCM group had the fewest transfused patients, but this difference did not reach significance when compared to FG.

Notably, basal ferritin levels revealed no significant differences between groups using both the mean value and the clinical cut-off (40 ng/mL) (Figure 1D).

The FCM group showed better iron restoration at the four week follow-up by comparing mean values of ferritin or by using the clinical cut-off (40 ng/mL) (Figure 1E).

Iron supplementation at 28 weeks matched with the ESA alone group and was effective for both treatment groups A and B (Figure 1F).

No side effects such as headache, nausea, and hypersensitivity reactions nor hypophosphatemia were reported among the whole population. A mild hypotension was reported in a patient treated with FG.

No cases of intolerance were reported in both iron supplemented groups.

Five patients in the FG group developed symptomatic phlebitis, which was treated with a local gel (1 g escin, 5000 U.I. heparin sodium, 5 g diethylamine salicylate) without sequelae.

FCM was revealed to be the cheapest treatment, followed by FG, while the most expensive treatment was ESAs (median expense, respectively, EUR 11.816 (11.816–23.480) vs. EUR 14.532 (14.532–14.532) vs. EUR 20.556 (20.556–20.556)), Table 2. This ranking was confirmed by Mann–Whitney test (for non-parametric data), finding a statistical difference between groups (*p* = 0.001 for each of the three comparisons).

## 4. Discussion

This paper suggests that iron replacement may be used to improve erythroid response and reduce RBCT for low-risk MDS patients receiving ESAs with or without iron deficiency. In this study, we have shown that patients manifested an effective and longer-lasting response to ESA treatment when they were supplemented with any type of iron.

When patients were supplemented with FCM, our data suggests a more rapid and long-lasting erythroid response, resulting in more rapid increase in hemoglobin, in a long-lasting hemoglobin stability, and in a reduction in number of RBCTs.

Historical concerns on iron supplementation in MDS syndrome arise from pathophysiology of MDS in which ineffective erythropoiesis may contribute, along with red blood cell transfusion, to iron overload.

In addition, iron levels have been considered for a long time a significant prognostic marker for relapse incidence of MDS and showed a significant positive correlation with the number of RBCTs. However, iron levels were not associated with progression to acute myeloid leukemia or with the time to transformation [18]. Higher iron levels were also an indicator of a lower likelihood of leukemia-free survival, relapse-free survival, and event-free survival [18]. Safety of parenteral iron is a historical concern that is based on experience from obsolete high molecular weight iron dextran formulations [19].

Our study suggests that FCM increases erythroid response and reduces the need of RBCT along with no immediate side effect such as rate of infection and iron overload, although more evidence is needed to confirm this result.

A potential bias in patient selection of our study can be overcome by the different times of enrollment according to iron formulation used and use in therapy. Given the observation in this study of prompt recovery of erythropoiesis with the supplementation of iron FCM and ESAs, it is reasonable to use FCM as a bridge before oral iron therapy.

A recent retrospective paper by Giordano et al. in a MDS population treated with ESA alone or with ESAs plus oral sucrosomial iron or IV FG (62.5 mg/week) confirmed this possibility [20]. Nonetheless, among the two studies, different doses of FG were administered. This dissimilarity could be explained by protocol differences: (1) a lower IV iron dose than ours (half); (2) an absent enrollment criterion about IPSS risk so that 46.7% of the study patients were IPSS class “Int-1”, while our patients were low-risk MDS (IPSS score = 0); (3) higher ferritin level prior iron supplementation (mean 634.89 ± 165.12 ng/mL); (4) enrolled patients with EPO more than 500 mU/mL (29.6% of the population).

In the literature, there is some limited evidence that intravenous iron is superior to oral iron on the basis of improvement in HB level. However, the results were not consistent across all other hematologic outcomes, and the quality of adverse outcomes reporting was poor. Comparison between oral and intravenous iron has been better studied in a systematic review and meta-analysis in patients with chronic kidney disease [21], which showed that patients treated with intravenous iron were more likely to reach an Hb response > 1 g/dL (risk ratios (RRs) of 1.61 (95% CI, 1.39–1.87) for chronic kidney disease stages 3–5, and 2.14 (95% CI, 1.68–2.72) for CKD stage 5D). Safety analysis showed similar rates of mortality and any adverse effects. Intravenous iron replacement was associated with higher risk for hypotension (RR, 3.71; 95% CI, 1.74–7.94) and fewer gastrointestinal adverse events (RR, 0.43; 95% CI, 0.28–0.67).

Nevertheless, the unresolved old question as to whether oral or intravenous iron therapy should be used still remains. Generally speaking, factors that should be considered in decision making include the patients’ age and sex, the underlying conditions, the cause of iron deficiency, the severity of anemia or ferritin values and their symptoms, and the time frame available or acceptable for correction. Intravenous therapy in MDS patients could be considered due to the weekly treatment with EPOs at high dose that requires weekly hospitalization.

For this reason and in order to provide a homogenous iron supplementation, we chose in this retrospective survey to consider intravenous iron administration instead of oral administration. FCM has been available for over a decade, is currently marketed in over 50 countries, and has been tested across a range of clinical indications [22].

Intravenous iron preparations also have the advantage of being able to deliver larger amounts of elemental iron in a single procedure and may also be more effective in patients with poor oral intake or absorption problems; moreover, it is not impacted by inflammation. The use of iron replacement in an oncological setting has been studied in three meta-analytic studies, demonstrating that, as compared with ESAs alone, iron replacement and ESAs increase the like hood of erythropoietic response [23,24,25].

In our retrospective case series, we confirmed the safety of intravenous iron and report no side effects with administration of FCM with only a slight hypotension with classic FG.

The economic evaluation of ESA treatment-related costs in MDS patients showed no increase in expenditures among patients and a possible cost saving, according to the type of iron supplementation they received. The more effective response and the lower need for ESA maintenance treatment recorded in the group treated with iron translated into a lowering of overall monthly expenditure per patient, 45% in the FCM group and 30% in the FG group. Indeed, although the treatment cycle with FCM had higher purchase costs compared to ferric gluconate, it greatly reduced other direct clinical expenditures such as minimizing hospital day numbers.

In this study, we opted to exclude the indirect costs for lost days of work, differing from the study of Giordano et al., due to the older age of patients enrolled and the difficulty of weighing this parameter on younger caregivers. Nevertheless, even if not included, the FCM group could benefit from this approach due to the lower hospital-day numbers.

Our study has several limitations: first, it was retrospective, and second, it included a low number of patients. The selection bias was overcome by the different timing of enrollments of the therapies, as confirmed by the homogeneity of the three groups at baseline analysis. Nevertheless, the results of this pilot study have to be confirmed in a future randomized control trial with a larger population. In addition, six months of study follow-up is a short time compared to the 5.7 years of median survival time associated in low-risk MDS. For these reasons, no information on course of the disease (e.g., evolution to AML, death); iron overload; and 6H syndrome secondary to FCM including osteomalacia, bone fractures, muscular weakness, and respiratory failure [26] could be assessed.

## 5. Conclusions

To the best of our knowledge, this is the first study that compared old and new types of intravenous iron supplementation with ESAs in low-risk MDS. We have shown that patients appeared to manifest a better and longer lasting response to ESA treatment when they were also supplemented with iron. Moreover, we confirmed the safety of intravenous iron, reporting no side effects. In the group treated with iron, an abatement of overall monthly expenditure per patient was seen.

## Figures and Tables

**Table 1:** Characteristic and laboratory exams of examined population and the three groups according to treatment. Continuous variables are expressed as “median (IQR)”.

	Patients Overall (*n* = 113)	(A) Patients with Ferric Gluconate and ESAs (*n* = 43)	(B) Patients with Ferric Carboximaltose and ESAs (*n* = 38)	(C) Patients with ESAs Alone (*n* = 32)	A vs. B	A vs. C	B vs. C
Gender							
Female	52 (42.3%)	18 (41.9%)	23 (60.5%)	11 (34.4%)	0.12	0.63	0.03
Male	71 (57.7%)	25 (58.1%)	15 (39.5%)	21 (65.6%)			
Age (years)	70 (66–74)	70 (68–74)	71.5 (66–76)	68 (66–72)	0.88	0.13	0.19
Basal hemoglobin (g/dL)	8.4 (8.2–8.5)	8.3 (8.2–8.5)	8.4 (8.2–8.7)	8.3 (8–8.5)	0.16	0.51	0.006
Basal hemoglobin (g/dL) > 9	10	3	6	1	0.29	1	0.12
Hemoglobin (g/dL) after 4 weeks (one month)	9.1 (8.6–9.5)	9.4 (9.1–9.6)	9.2 (8.6–9.5)	8.5 (8–8.7)	0.22	<0.001	<0.001
Hemoglobin (g/dL) > 9 after 4 weeks (one month)	58	33	23	2	0.15	<0.001	<0.001
Hemoglobin (g/dL) after 28 weeks (six months)	11.4 (10.5–12)	12 (12–12.2)	10.2 (9.2–11.1)	8.7 (8.1–10)	<0.001	<0.001	<0.001
Hemoglobin (g/dL) > 10 after 28 weeks (six months)	81	41	31	9	0.8	<0.001	<0.001
Number of transfusions	37	8	4	25	0.36	<0.001	<0.001
Number of transfusions during the first 4 weeks	27	8	4	15	0.36	0.012	0.001
Basal ferritin (ng/mL)	12 (6–30)	6 (4–12)	30 (10–40)	14 (10–30)	0.096	1	0.379
Basal ferritin (ng/mL) > 40	29	7	11	11	0.19	0.1	0.79
Ferritin (ng/mL) after 4 weeks (one month)	23 (14–48)	20 (14–40)	48 (20–69)	20 (12–28)	0.11	0.186	0.001
Ferritin (ng/mL) > 40 after 4 weeks (one month)	58	15	25	9	0.008	0.62	0.002
Ferritin (ng/mL) after 28 weeks (six months)	60 (40–120)	60 (40–140)	80 (57–131)	42 (26–60)	1	0.007	0.019
Ferritin (ng/mL) > 40 after 28 weeks (six months)	91	37	32	18	1	0.007	0.016

**Table 2:** Direct and indirect treatment-related cost (EUR) items. Data are reported as “median (IQR)”.

	Patients Overall (*n* = 113)	(A) Patients with Ferric Gluconate and ESAs (*n* = 43)	(B) Patients with Ferric Carboximaltose and ESAs (*n* = 38)	(C) Patients with ESAs Alone (*n* = 32)	A vs. B	A vs. C	B vs. C
Day hospital	299.000	240.800	53.200	5.000			
Transfusion	6.660	1.440	720	4.500			
Iron	90.412	18.060	72.352	0			
EPO	1.555.168	481.104	451.760	622.304			
Total cost	1.951.240	741.404	578.032	631.804			
Expenditure for patient	14.532 (11.816–20.556)	14.532 (14.532–14.532)	11.816 (11.816–23.480)	20,556 (20,556–20,556)	*p* = 0.001	*p* = 0.001	*p* = 0.001
Four weeks (one month) expenditure for patient	2076 (1688–2937)	2076 (2076–2076)	1688 (1688–3354)	2.937 (2937–2937)	*p* = 0.001	*p* = 0.001	*p* = 0.001
One-week expenditure for patient	519 (422–734)	519 (519–519)	422 (422–829)	734 (734–734)	*p* = 0.001	*p* = 0.001	*p* = 0.001

## Data Availability

The study data will be made available upon request to the corresponding author.
